# Altered Low-beta Characteristics in Individuals With Alcohol Use Disorder: A Pilot Resting Electroencephalography Study

**DOI:** 10.31083/AP40025

**Published:** 2025-12-19

**Authors:** Bing Li, Jie Wang, Shuaiyu Long, Jinyun Hu, Lili Zhang, Wei Cui, Yunshu Zhang, Chaomeng Liu

**Affiliations:** ^1^Hebei Provincial Mental Health Center, 071000 Baoding, Hebei, China; ^2^Department of Psychiatry, The Sixth Clinical Medical College of Hebei University, 071000 Baoding, Hebei, China; ^3^The National Clinical Research Center for Mental Disorders & Beijing Key Laboratory of Mental Disorders, Beijing Anding Hospital, Capital Medical University, 100088 Beijing, China; ^4^Advanced Innovation Center for Human Brain Protection, Capital Medical University, 100054 Beijing, China

**Keywords:** alcohol use disorder, electroencephalography, dynamic functional connectivity, k-means clustering

## Abstract

**Background:**

The pathophysiological mechanisms underlying alcohol use disorder (AUD) remain unclear, and its clinical evaluation largely depends on subjective assessments lacking objective biomarkers. This study employed a case-control design incorporating resting-state electroencephalography (EEG) with power spectral analysis (PSA) and dynamic functional connectivity (dFC) to explore potential biomarkers for AUD.

**Methods:**

Resting-state EEG data were collected from individuals diagnosed with AUD and demographically matched healthy controls (HCs), alongside comprehensive neuropsychological and behavioral evaluations. PSA quantified energy distribution across specific frequency bands, with receiver operating characteristic analysis determining its discriminatory capacity. dFC was examined using a sliding window approach and the weighted phase-lag index, followed by K-means clustering to extract dominant connectivity states across frequency bands.

**Results:**

After excluding cases with suboptimal EEG data, the final analytic sample comprised 25 individuals with AUD and 26 HCs. Compared to HCs, the AUD group exhibited elevated low-beta power at F1, FCz, FC1, and C3 electrode sites (10-20 EEG system), with respective area under the curve values of 0.795, 0.794, 0.806, and 0.769, indicating reliable group differentiation. Temporal profiling of functional connectivity revealed three distinct brain states: S1 (60.81%), S2 (21.05%), and S3 (18.15%). Correlations between these connectivity patterns and clinical indices were observed in the AUD cohort.

**Conclusion:**

Individuals with AUD showed increased brain activity in the medial frontal gyrus and left central gyrus at rest, as well as significant low-beta frequency changes in dFC analysis. Resting EEG scans with PSA and dFC analysis could serve as potential biomarkers for detecting AUD.

## Main Points

∙ In individuals with alcohol use disorder (AUD), power spectral analysis indicated increased low-beta power at F1, FCz, FC1, and C3 electrodes relative to healthy controls, enabling effective differentiation between groups;

∙ Whole-brain functional connectivity across all time windows was classified into three discrete states via k-means clustering, comprising 60.81%, 21.05%, and 18.15% of the data, respectively;

∙ Correlational analysis identified associations between the brain state index and both the obsessive thoughts of drinking and social subscale scores within the AUD cohort.

## 1. Introduction

Alcohol use disorder (AUD) is a chronic, relapsing condition marked by persistent excessive alcohol consumption despite significant health and social consequences [1, 2]. To date, the underlying mechanisms of addiction and relapse remain incompletely understood. Additionally, diagnostic and evaluative approaches for AUD predominantly rely on self-reported questionnaires, which may be prone to bias and deception [3]. Incorporating objective biomarkers into the evaluation of alcohol dependence could reinforce the conceptual framework of addiction and enhance both diagnostic precision and therapeutic strategies.

Resting-state electroencephalography (EEG), which captures intrinsic neural activity in the absence of task demands, generates oscillatory electrical signals that can be characterized by their frequency, amplitude, spectral distribution, and phase dynamics [4, 5]. These signals are typically categorized into frequency bands [6] and power spectral analysis (PSA) measures the energy in these rhythms, and differences in PSA between individuals with AUD and healthy controls (HCs) may highlight AUD’s pathology. Studies show individuals with AUD have higher theta energy [7], possibly indicating impaired information encoding [8]. Compared to gamma activity, beta-band dynamics have undergone more extensive investigation; whole-brain beta power is consistently elevated in AUD, independent of demographic or clinical variables such as age, alcohol intake patterns, and timing of assessment, with stronger effects reported in males [9, 10]. Recent advancements propose integrating machine learning algorithms with resting-state EEG and synchronization likelihood metrics to discriminate AUD from HCs, offering a framework for automated EEG-based diagnostic systems [11]. While PSA enables regional brain activity analysis, comprehensive understanding requires examination of large-scale functional network architecture. While PSA enables regional brain activity analysis, comprehensive understanding requires examination of large-scale functional network architecture. Connectivity assessments employing coherence, synchronization, and phase-locking methodologies facilitate the interrogation of inter-regional communication via multichannel electrode arrays [3]. A reported enhancement in theta coherence (6–7 Hz) among AUD subjects has been hypothesized to relate to craving-related processes [12].

Through initial searches of the PubMed database, we have identified that the understanding of EEG characteristics in AUD primarily originates from studies that consider functional connectivity (FC) as a temporally invariant construct.

However, the brain inherently operates as a dynamic system, persistently reorganizing its connectivity to accommodate internal and external perturbations [13]. To overcome the methodological constraints of static models, recent investigations have explored dynamic functional connectivity (dFC) paradigms [14]. Evidence from these studies indicates that the brain cycles through transient FC patterns, reflecting spontaneous fluctuations in cognitive states even during rest [15, 16]. Despite these advances, EEG-based dFC in the context of AUD remains unclear, leaving critical aspects of the disorder’s neural dynamics unexplored.

In this study, spectral power differences across multiple frequency bands of resting-state EEG signals were examined between age- and sex-matched individuals with AUD and healthy controls. A sliding window strategy, integrated with the weighted phase-lag index, was employed to estimate intra-channel dFC. K-means clustering was subsequently applied to identify distinct connectivity states across the examined frequency bands. Statistically significant state-related features were then subjected to correlation analysis with clinical measures.

## 2. Methods

### 2.1 Participants

Individuals diagnosed with AUD and HCs were enrolled from the Hebei Provincial Mental Health Center (Baoding, China) between March 2022 and December 2023. The inclusion and exclusion criteria of study participants are presented in **Supplementary Material 1**.

### 2.2 Neuropsychological and Behavioral Assessment 

All participants completed a comprehensive clinical evaluation following enrollment, incorporating the Alcohol Use Disorders Identification Test (AUDIT) [17], the Clinical Institute Withdrawal Assessment for Alcohol-Revised (CIWA-Ar) scale [18], and the Obsessive-Compulsive Drinking Scale (OCDS) [19], which includes subscales targeting obsessive drinking thoughts (OB) and compulsive drinking behavior (CP). The Drinking Motives Questionnaire-Revised (DMQ-R) [20], including four dimensions—Social (SOC), Enhancement (ENH), Coping (COP), and Conformity (CON)—was administered to assess individual drinking motivations.

The recording and preprocessing of the EEG signals are detailed in **Supplementary Material 2**. Furthermore, comprehensive descriptions of the PSA and dFC methodologies are provided in **Supplementary Material 3**. The brain regions associated with the EEG channels were localized by the identifying the following 12 regions of interest **Supplementary Fig. 1**.

### 2.3 Statistical Analysis

Statistical analysis was conducted using SPSS version 24.0 (IBM SPSS Corp., Armonk, NY, USA), with significance set at *p* < 0.050. For non-normally distributed variables (e.g., years of education), data were summarized as median (25th percentile, 75th percentile), whereas normally distributed variables (e.g., age) were reported as mean ± SD. Group comparisons between AUD and HC participants were performed using Welch’s two-sample *t*-test, Wilcoxon rank-sum test, or Fisher’s exact test, depending on data type and distribution.

Power spectral differences across EEG channels were evaluated using independent-sample *t*-tests with family-wise error (FWE) correction. Power in distinct frequency bands was calculated using MATLAB 2023a (MathWorks, Natick, MA, USA). The diagnostic capacity of PSA outcomes was quantified via receiver operating characteristic (ROC) curve analysis; greater area under the curve (AUC) values reflected superior discriminative performance. Youden’s J statistic (sensitivity + specificity – 1) was employed to identify the optimal cutoff, defined as the point of maximum J value [21]. dFC differences were examined with independent-sample *t*-tests and FWE correction using the GRETNA (v2.0.0) toolbox (http://www.nitrc.org/projects/gretna/). Whole-brain functional connectivity time windows were clustered into three brain states through k-means clustering. For each participant, state frequency, mean dwell time, number of transitions, and transition matrices across frequency bands were computed. Associations between these brain state metrics and clinical measures within the AUD group were assessed using Spearman’s correlation analysis.

## 3. Results

### 3.1 Demographics and Neuropsychologic Data

Initially, 27 individuals diagnosed with AUD and 27 HCs were included in the study. However, after excluding cases with suboptimal EEG data, the final analytical sample consisted of 25 individuals with AUD and 26 HCs. The final analysis comprised 25 male participants with AUD and 26 male HCs. Compared with the HC group, the AUD group demonstrated markedly lower educational attainment (9.00 [9.00, 12.00] vs. 14.00 [12.00, 16.00], *p* < 0.001). Significant group differences were also observed in multiple behavioral parameters. The AUD group reported longer alcohol use duration (21.20 ± 6.70 vs. 14.04 ± 12.09 years, *p* = 0.012), greater daily drinking frequency (3.00 [2.00, 6.00] vs. 1.00 [1.00, 1.00], *p* < 0.001), and higher daily alcohol consumption (225.00 [180.00, 270.00] g vs. 54.00 [19.50, 90.00] g, *p* < 0.001). Tobacco-related indices were also significantly elevated, including smoking duration (20.00 [17.50, 26.50] vs. 0.00 [0.00, 20.00] years, *p* = 0.001) and cigarettes per day (20.00 [15.00, 25.00] vs. 0.00 [0.00, 12.50], *p* < 0.001). AUDIT (23.00 [17.00, 33.00] vs. 4.00 [2.00, 5.25], *p* < 0.001), CIWA-Ar (2.00 [1.00, 4.50] vs. 0.00 [0.00, 0.00], *p* < 0.001), and OCDS (21.00 [16.50, 25.00] vs. 1.00 [0.00, 3.00], *p* < 0.001) scores were all significantly elevated in the AUD group. Subscale analysis further revealed higher scores across OB (8.00 [6.25, 12.00] vs. 0.00 [0.00, 0.00], *p* < 0.001), CP (11.00 [8.50, 14.50] vs. 1.00 [0.00, 3.00], *p* < 0.001), ENH (12.00 [8.00, 18.50] vs. 5.00 [5.00, 6.25], *p* < 0.001), COP (10.00 [8.00, 15.00] vs. 5.00 [5.00, 6.25], *p* < 0.001), and CON (8.00 [6.00, 10.00] vs. 6.00 [5.00, 8.25], *p* = 0.026). A comprehensive summary of demographic, neuropsychological, and behavioral assessments stratified by group was provided in Table 1.

**Table 1.** **Characteristics of the alcohol use disorder group and healthy control group**.

Variables		AUD group (n = 25)	HC group (n = 26)	Statistics (*t/Z*)	*p*-value
Age (years)		44.16 (5.89)	39.19 (13.26)	1.74	0.091^1^
Years of Education		9.00 [9.00, 12.00]	14.00 [12.00, 16.00]	3.84	<0.001^2^
Sleep_duration (hours)		6.50 [6.00, 8.00]	7.00 [6.88, 8.00]	1.68	0.093^2^
Drinking years		21.20 (6.70)	14.04 (12.09)	2.63	0.012^1^
Drinking times (per day)		3.00 [2.00, 6.00]	1.00 [1.00, 1.00]	6.05	<0.001^2^
Alcohol consumption (g/d)		225.00 [180.00, 270.00]	54.00 [19.50, 90.00]	5.89	<0.001^2^
Smoking years		20.00 [17.50, 26.50]	0.00 [0.00, 20.00]	3.29	0.001^2^
Cigarette Consumption (n/d)		20.00 [15.00, 25.00]	0.00 [0.00, 12.50]	4.04	<0.001^2^
AUDIT		23.00 [17.00, 33.00]	4.00 [2.00, 5.25]	6.19	<0.001^2^
CIWA-Ar		2.00 [1.00, 4.50]	0.00 [0.00, 0.00]	6.07	<0.001^2^
OCDS		21.00 [16.50, 25.00]	1.00 [0.00, 3.00]	5.72	<0.001^2^
	OB	8.00 [6.50, 12.00]	0.00 [0.00, 0.00]	6.07	<0.001^2^
	CP	11.00 [8.50, 14.50]	1.00 [0.00, 3.00]	5.72	<0.001^2^
SOC		11.00 [8.50, 12.50]	8.50 [6.00, 12.00]	1.94	0.052^2^
ENH		12.00 [8.00, 18.50]	5.00 [5.00, 6.25]	4.65	<0.001^2^
COP		10.00 [8.00, 15.00]	5.00 [5.00, 6.25]	5.02	<0.001^2^
CON		8.00 [6.00, 10.00]	6.00 [5.00, 8.25]	2.22	0.026^2^

**Note**: Mean (SD); Median [25th percentile, 75th percentile]; ^1^Two Sample *t*-test; ^2^Mann-Whitney U test; AUD, Alcohol use disorder; HC, healthy control; AUDIT, the Alcohol Use Disorders Identification Test; CIWA-Ar, the Clinical Institute Withdrawal Assessment-Alcohol; OCDS, the Obsessive-Compulsive Drinking scale; OB, obsessive thoughts of drinking subscale; CP, compulsive drinking subscale; SOC, Social score; ENH, Enhancement score; COP, Coping score; CON, Conformity score.

### 3.2 Group Differences in PSA and ROC Curve Analysis

**Fig. 1.**
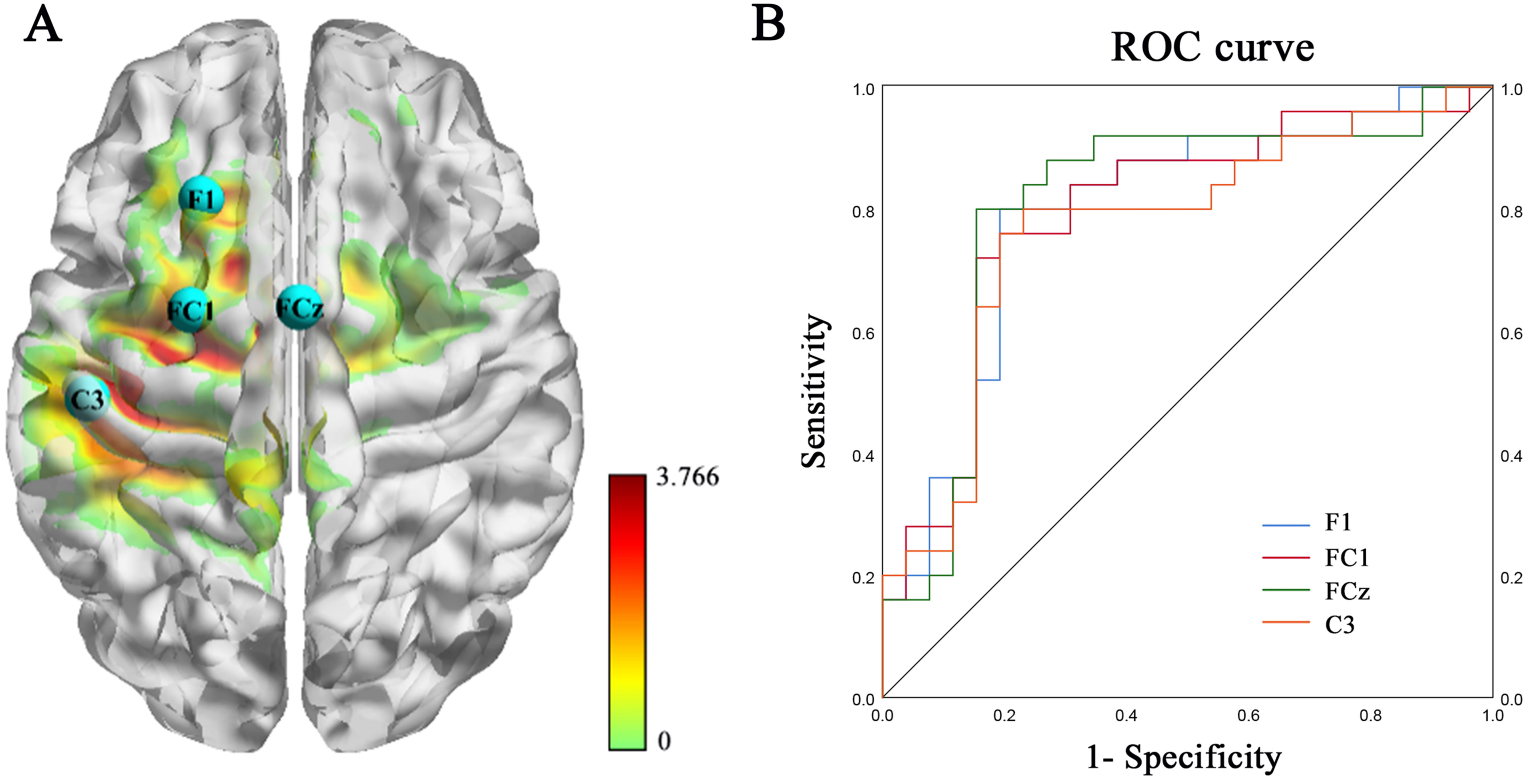
**Results of Power spectral analysis and receiver operating characteristic curve analysis under eyes-closed conditions**. (A) Electrodes with significantly different power in the power spectral analysis between groups. (B) Receiver operating characteristic curve analysis of power of F1, FCz, FC1, and C3 electrodes in distinguishing alcohol use disorder group from healthy control group. The topology diagram displays the analysis results at the electrode level, and the color represents the interpolation effect. ROC, receiver operating characteristic.

PSA indicated significantly elevated low-beta band power in the AUD group relative to the HC group at F1 (*t *= 3.701, *p* = 0.001), FC1 (*t *= 3.766, *p* < 0.001), FCz (*t* = 3.650, *p* = 0.001), and C3 (*t* = 3.741, *p* < 0.001) electrodes during the eyes-closed condition, primarily implicating the middle frontal gyrus (MFG) and left central gyrus (LCG) (Fig. 1A). No significant group differences were detected across other frequency bands. Discriminative capacity of spectral power at the above sites was further assessed using ROC curve analysis. The resulting curves exhibited comparable trajectories (Fig. 1B). AUC values for F1, FC1, FCz, and C3 were 0.795 (95% confidence interval (CI): 0.668–0.923, sensitivity: 0.800, specificity: 0.808), 0.794 (95% CI: 0.665–0.922, sensitivity: 0.760, specificity: 0.808), 0.806 (95% CI: 0.675–0.938, sensitivity: 0.800, specificity: 0.846), and 0.769 (95% CI: 0.635–0.904, sensitivity: 0.800, specificity: 0.769), respectively (Table 2).

**Table 2.** **Receiver operating characteristic curve analysis of power values of altered electrodes in distinguishing the alcohol use disorder group from the healthy control group under eyes-closed conditions**.

Power	AUC, 95% CI	Sensitivity, %	Specificity, %	Cut off point
F1	0.795 (0.668–0.923)	0.800	0.808	0.968
FC1	0.794 (0.665–0.922)	0.760	0.808	0.951
FCz	0.806 (0.675–0.938)	0.800	0.846	0.967
C3	0.769 (0.635–0.904)	0.800	0.769	0.867

**Note**: AUC, area under curve; CI, confidence interval.

### 3.3 Group Differences in dFC and Clustering Analysis

**Fig. 2.**
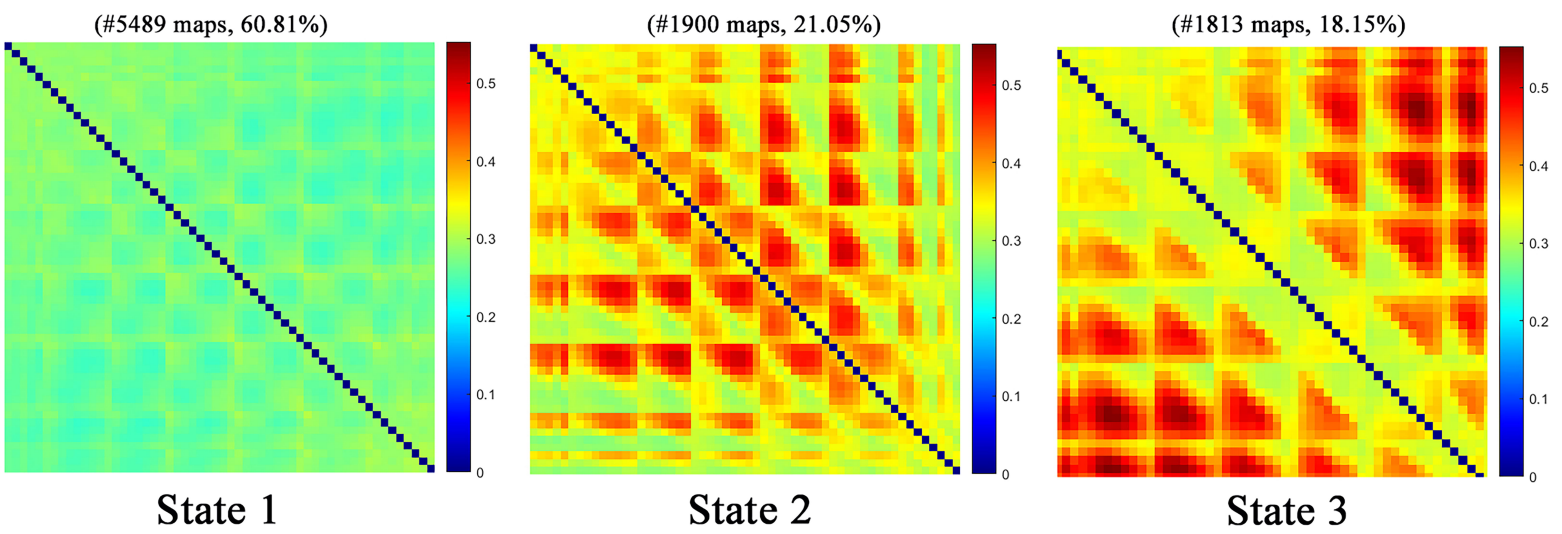
**Results of k-means clustering analysis under eyes-closed conditions**. The whole brain functional connectivity of all windows of all participants was clustered into three states by k-means clustering method.

**Fig. 3.**
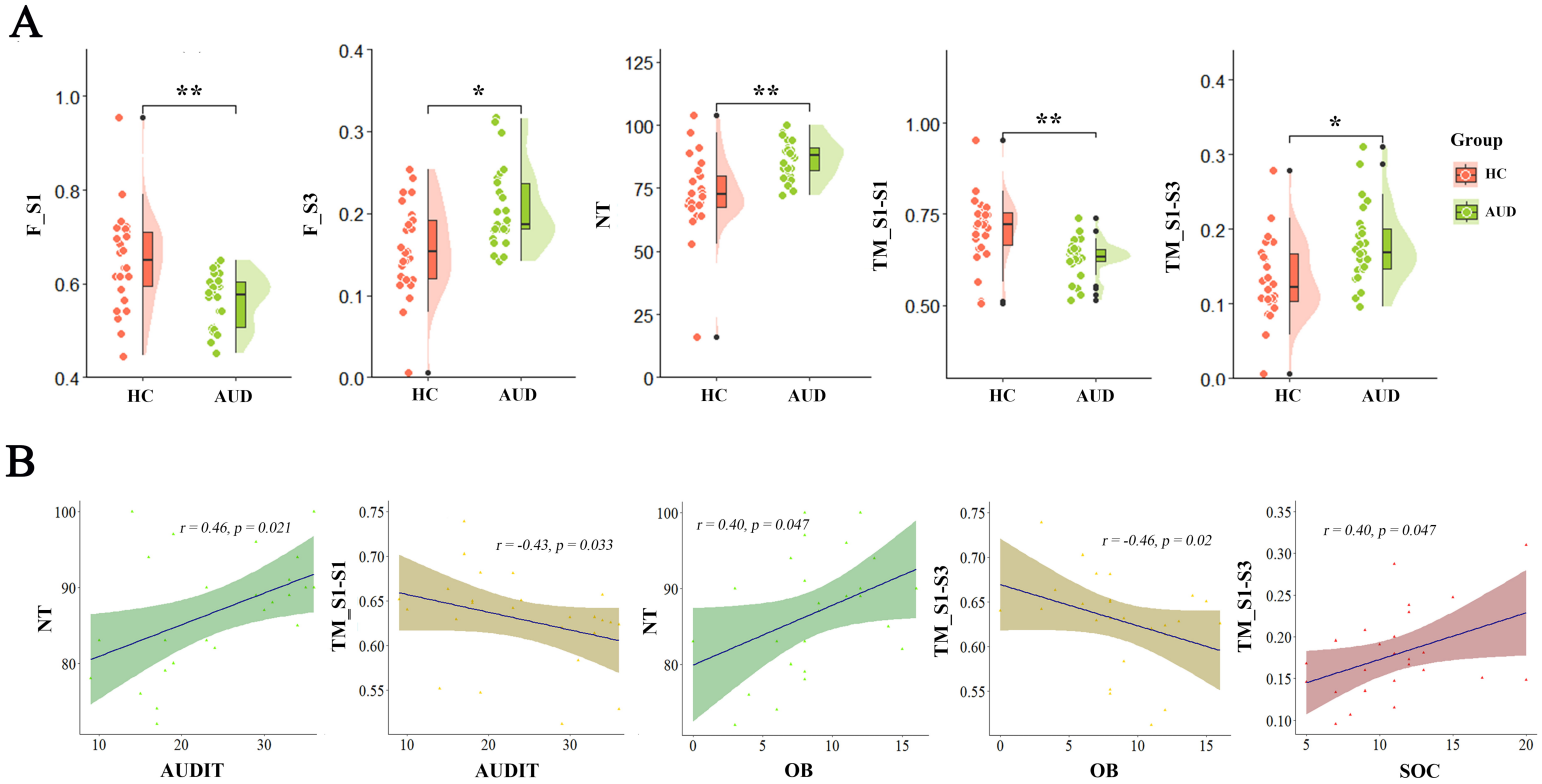
**Comparisons of brain state parameters and correlation analysis under eyes-closed conditions**. (A) Comparisons of brain state parameters between groups under eyes-closed conditions. (B) Correlation analysis between statistically significant brain state features and clinical characteristics in the alcohol use disorder group. F_S1, Frequency of State 1; F_S3, Frequency of State 3; NT, number of transitions between the three states; TM_S1–S1, TM, transition matrix, representing the transition probability between state 1 to state 1; TM_S1–S3 represented the transition probability between state 1 to state 3. *p < 0.05; **p < 0.001.

Consistent with PSA outcomes, significant group-level disparities emerged exclusively within the low-beta frequency band. Brain states S1, S2, and S3, identified through clustering analysis in this band, were depicted in Fig. 2. A decreasing trend in occurrence was observed across the states—S1 (60.81%), S2 (21.05%), and S3 (18.15%)—while connection density exhibited a progressive increase from S1 to S3. Comparative analysis of state occurrence between groups revealed a markedly lower frequency of S1 in the AUD cohort relative to HCs (*t *= –3.609, *p* < 0.001), whereas S3 occurred significantly more often among AUD participants (*t *= 3.328, *p* = 0.002). Additionally, AUD individuals exhibited a higher total number of state transitions (*t* = 3.888, *p* < 0.001). Analysis of the transition matrix further indicated a diminished self-transition probability for S1 in the AUD group (*t* = –3.807, *p* < 0.001), alongside an increased likelihood of transitioning from S1 to S3 (*t *= 3.085, *p* = 0.003), relative to the HC group (Fig. 3A).

### 3.4 Correlation Analysis

Correlation analysis indicated a significant association between AUDIT scores and dynamic connectivity features: higher AUDIT scores corresponded to increased transition counts (*r* = 0.46, *p* = 0.021) and decreased self-transitions within state S1 (*r* = –0.43, *p* = 0.033). Similarly, OB subscale scores demonstrated a positive relationship with the total number of transitions (*r* = 0.40, *p* = 0.047) and an inverse relationship with transitions from S1 to S3 (*r* = –0.46, *p* = 0.020). In contrast, SOC subscale scores were positively associated with transitions from S1 to S3 specifically within the AUD group (*r* = 0.40, *p* = 0.047) (Fig. 3B).

## 4. Discussion

This study employed resting-state EEG to investigate neural alterations in AUD by integrating PSA with dFC analysis. Compared to HCs, individuals with AUD demonstrated elevated low-beta power at F1, FC1, FCz, and C3 electrodes, corresponding to the MFG and LCG regions under eyes-closed condition. These channels yielded substantial discriminative capacity between groups, with AUCs indicative of effective group classification. Cluster-based assessment of low-beta dFC dynamics identified group-specific variations in frequency, number of transitions, and transition matrix of three different brain states. Moreover, these differences were related to the neurobehavioral performance of individuals with AUD.

Elevated low-beta power has been proposed as a neurophysiological marker of cortical hyperexcitability, likely reflecting disrupted excitation—inhibition homeostasis in individuals with AUD [9, 22]. Chronic alcohol exposure may further intensify this dysregulation, reinforcing the neural basis of impaired inhibitory control. Theoretical frameworks of AUD conceptualize deficient cognitive regulation as both a predisposing vulnerability and a downstream effect of sustained alcohol use [23, 24]. Accumulating evidence supports the role of oscillatory dynamics, particularly theta and beta band activity within the MFG, in mediating cognitive control processes [25]. Prior investigations have demonstrated that anodal transcranial direct current stimulation enhances inhibitory capacity [26], a modulation attributed to altered medial-frontal cortical activity and strengthened functional coupling between the presupplementary motor area and the ventromedial prefrontal cortex. Furthermore, the LCG has been implicated in processing aversive feedback [27] and plays an essential role in governing response inhibition [28]. PSA alterations localized to the MFG and LCG suggest functional disruption in these regions, implicating alcohol-induced neurotoxicity in the deterioration of executive inhibition. Future therapeutic protocols may consider targeting the F1, FC1, FCz, and C3 electrode sites to optimize neuromodulatory interventions for AUD.

The present investigation was limited to male participants. Preclinical evidence indicates that voluntary alcohol intake during adolescence reduces the density of myelinated axons within the anterior cingulate subregion of the medial prefrontal cortex [29]. Adolescent alcohol exposure has also been linked to impaired adult working memory performance [29]. Notably, such myelin density reductions were absent in female rats subjected to adolescent binge drinking [30], consistent with murine data showing a less significant decline in myelin gene expression in adolescent females relative to males following high-dose alcohol exposure [31]. These findings collectively point to a sex-specific vulnerability, with male cortical circuits implicated in executive and behavioral regulation appearing more susceptible to alcohol-related alterations. Enhancing female representation in AUD research and incorporating sex as a biological variable may refine insights into the neurobiological basis of AUD. Additionally, participants with AUD in this study had lower educational attainment than HCs. Prior research suggests that higher education may offer a protective effect against cognitive deterioration in AUD populations [32]. Thus, in AUD, reduced cognitive function may arise not only from alcohol-related neuropathology but also from lower baseline educational levels. Supporting this, EEG study has demonstrated that elevated high-beta band (25–30 Hz) oscillatory activity correlates with increased risk for mild cognitive impairment [33]. Future investigations should account for educational disparities as a potential confounding factor influencing cognitive outcomes.

Subjective scales remain the predominant tools in clinical settings for quantifying alcohol craving; however, the lack of objective indices limits accurate characterization of craving intensity and impedes individualized intervention. ROC analysis revealed that spectral power at F1, FCz, FC1, and C3 electrodes yielded high discriminative performance, as reflected by robust AUC values distinguishing the two cohorts. A recent study by Mohd Nazri *et al*. [34] applied partial directed coherence analysis to resting-state EEG data, integrating SVM classifiers for AUD identification, achieving 94.6% classification accuracy with an AUC above 0.98. This method demonstrates strong diagnostic potential as a non-invasive alternative for differentiating AUD participants from HCs. Subsequent investigations may refine this framework by exploring associations between EEG channel energy metrics and craving severity, contributing to longitudinal disease monitoring and adaptive treatment strategies.

Correlation analysis indicated that higher AUDIT scores were associated with a greater number of state transitions and reduced self-transition probability within S1, suggesting that elevated alcohol dependence severity may correspond to increased instability in brain functional dynamics among individuals with AUD. The OB subscale score demonstrated a similar pattern—positively correlated with transition count and inversely related to transitions from S1 to S3—indicating that intensifying compulsive drinking ideation may coincide with weakened inter-state connectivity. Additionally, the SOC subscale score of the DMQ-R correlated positively with transitions from S1 to S3, implying that stronger social motivation may correspond to greater connectivity within the brain’s functional network in AUD. It should be noted that although correlations are established between brain state indices and clinical scale scores within the AUD group, the incorporation of EEG data from healthy controls during clustering may have introduced heterogeneity into the connectivity state definition. Future analyses will aim to refine methodological rigor by addressing this limitation.

Several limitations warrant consideration. The small sample size and exclusive inclusion of male participants may restrict the applicability of the results to broader populations. Additionally, the absence of a standardized protocol for determining sliding window length in dFC analysis introduces methodological variability; variations in window size could influence the stability and sensitivity of connectivity estimates. Moreover, although source localization is commonly applied prior to connectivity computation to address confounding effects such as volume conduction and reference electrode bias [35], the present study prioritizes characterizing the temporal dynamics of EEG-derived connectivity patterns in AUD, including state proportions, durations, and transition probabilities. Future investigations are planned to incorporate individualized source reconstruction techniques to enhance data standardization and spatial accuracy.

## 5. Conclusion

In conclusion, individuals with AUD exhibited increased brain activity in the MFG and LCG at rest, as well as significant low-beta frequency changes in dFC analysis across brain regions. Resting EEG scans, utilizing PSA and dFC analysis, could aid in identifying potential biomarkers for the detection of AUD.

## Availability of Data and Materials

The data that support the findings of this study are available from the corresponding author upon reasonable request.

## Supplementary Material

Supplementary material associated with this article can be found, in the online version, at https://doi.org/10.31083/AP40025.
