# Supplementary material for: Altered Low-beta Characteristics in Individuals With Alcohol Use Disorder: A Pilot Resting Electroencephalography Study
Source: Alpha Psychiatry. 2025 Dec 19;26(6):40025. doi: 10.31083/AP40025 (PMC12781204; doi:10.31083/AP40025)
Supplement: Supplementary file 1 [file 2757-8038-26-6-40025-s1.docx]

Supplementary Information for

Altered low-beta characteristics in individuals with alcohol use disorder: A pilot resting electroencephalography study

**Supplementary Material 1**

***Inclusion and exclusion criteria***

Eligibility criteria included Han Chinese ethnicity, right-handedness, age between 18 and 60 years, and the capacity to complete clinical assessments. Diagnostic evaluation was conducted by an experienced psychiatrist using the Structured Clinical Interview for DSM-5 (SCID) for DSM-5, with AUD confirmed in individuals meeting DSM-5 criteria for alcohol dependence.^1^ All AUD participants had maintained abstinence from alcohol for a minimum of two weeks before participation, verified through both self- and caregiver reports, which were deemed reliable based on regular outpatient follow-up and compliance with treatment regimens.^2^ AUD severity was quantified using the Alcohol Use Disorders Identification Test (AUDIT), which assesses hazardous use, dependence-related features, and harmful drinking behavior.^3^ Exclusion criteria included inability to provide informed consent, intellectual disability, significant systemic illnesses (e.g., severe cardiovascular, hepatic, or renal pathology), pregnancy or lactation, and history of alternative antipsychotic treatment. Comprehensive evaluations—including breath alcohol testing, physical examination, neuropsychological batteries, and EEG—were completed for all participants. HCs were locally recruited, matched for age, sex, and education, and screened to exclude any DSM-5 Axis I disorders.

**Supplementary Material 2**

***Recording and preprocessing of the EEG signals***

Participants were seated in a sound-attenuated, isolated environment adjacent to a recording room separated by a one-way glass window and remained in a resting state throughout data acquisition. EEG signals were collected using the 10–05 electrode placement system over a 10-minute session, divided equally between eyes-open and eyes-closed conditions. A 64-channel Quik-cap system (Compumedics Neuroscan) recorded EEG signals at a sampling rate of 1000 Hz. Visual inspection revealed substantial signal degradation under the eyes-open condition; therefore, subsequent analyses utilized only data from the eyes-closed segment. Preprocessing of the raw EEG data was conducted in MATLAB 2023a (The MathWorks, Natick, MA, USA) using the EEGLAB toolbox.^4^ The process involved applying the standard electrode template for channel localization, excluding channels exhibiting negligible EEG activity,^5^ and re-referencing signals to the bilateral mastoids. Additional preprocessing steps included bandpass filtering (0.1–45 Hz), segmentation into 2-second epochs,^6^ interpolation, and removal of channels with incomplete or corrupted data.^7^ Independent component analysis (ICA) was then applied to isolate and remove artifacts related to ocular and muscular activity, retaining components indicative of neural activity.

**Supplementary Material 3**

***Methodology of power spectral analysis and dynamic functional connectivity analysis***

For the preprocessed resting-state EEG data, given the existing research findings on the electrophysiological signals associated with AUD,^8^ the EEG signals were divided into six specific frequency bands, including delta (0.5–5 Hz), theta (5–8 Hz), alpha (8–13 Hz), low-beta (13–20 Hz), high-beta (20–35 Hz), and gamma (35–45 Hz). PSA of each frequency band at the whole-brain channels was conducted, followed by dFC analysis. According to previous research using PSA,^9^ the brain regions associated with the EEG channels were localized by the identifying the following 12 regions of interest (ROIs): (1) left frontal (LF) region comprising Fp1, AF3, F7, F5, and F3; (2) medial frontal (MF) region including F1, Fz, F2, FC1, FCz, and FC2; (3) right frontal (RF) region consisting of F4, Fp2, AF4, F6, and F8; (4) left temporal (LT) region with T7 and TP7; (5) left central (LC) region containing FC5, FC3, C5, C3, CP5, and CP3; (6) medial central (MC) region involving C1, Cz, C2, CP1, CPz, and CP2; (7) right central (RC) region comprising FC4, FC6, C4, C6, CP4, and CP6; (8) right temporal (RT) region with T8 and TP8; (9) left parietal (LP) region including P7, P5, P3, PO7, and PO3; (10) medial parietal (MP) region consisting of P1, Pz, P2, and POz; (11) right parietal (RP) region containing P4, P6, P8, PO4, and PO8; and (12) occipital (O) region with O1, Oz, and O2 (**S-figure 1**).


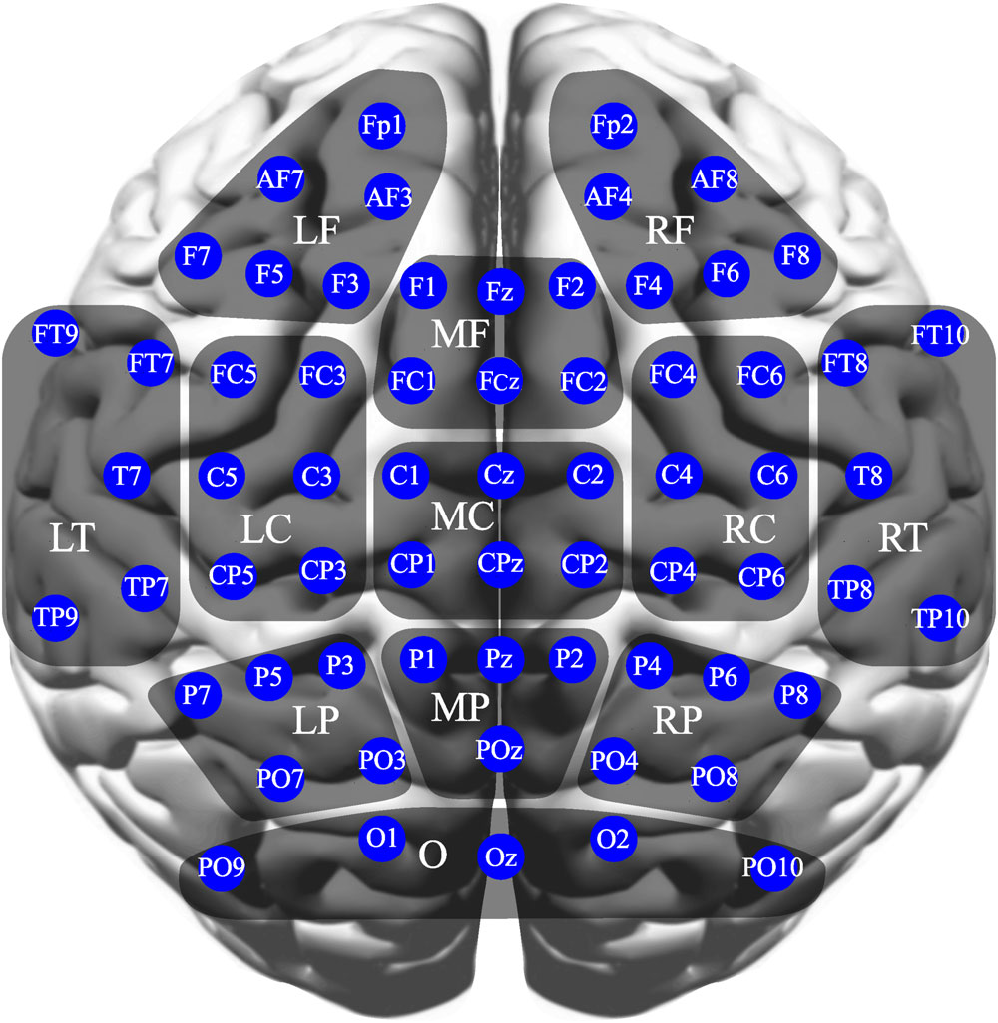


**Supplementary Fig. 1.** The 12 regions of interest. LC, left central; LF, left frontal; LP, left parietal; LT, left temporal; MC, medial central; MF, medial frontal; MP, medial parietal; O, occipital; RC, right central; RF, right frontal; RP, right parietal; and RT, right temporal.

In the dFC analysis, a sliding window of 2 s with a step size of 1 s was used. The weighted phase-lag index (wPLI) of each channel pair in each window was then calculated as the functional connectivity value. The whole-brain functional connectivity of all windows of all participants was clustered into three brain states by k-means clustering using the dynamic brain connectome analysis toolbox (https://guorongwu.github.io/DynamicBC/). Frequency (F), mean dwell time (MDT), number of transitions (NT), and transition matrix (TM) of each brain state were calculated using a home-written program in MATLAB.

# K-means clustering,^10^ a widely utilized algorithm in data analysis, identifies optimal categorical attributes by assessing the similarity between data points. The algorithm initiates by selecting \( k \) initial cluster centers. Subsequently, it computes the distance between each data point and these \( k \) centers, assigning each point to the nearest cluster based on the principle of minimum distance, specifically employing the Euclidean distance metric in this study. The mean of each cluster is then calculated and designated as the new cluster center. This iterative process continues until the cluster centers stabilize or a predefined maximum number of iterations is reached. In the context of DynamicBC's k-means clustering analysis, users are advised to select a cluster number ranging from 1 to 6. The toolbox then automatically determines and outputs the optimal number of clusters, thereby facilitating effective data categorization.

# Supplementary References

1. Lobbestael J, Leurgans M, Arntz A. Inter-rater reliability of the Structured Clinical Interview for DSM-IV Axis I Disorders (SCID I) and Axis II Disorders (SCID II). Clin Psychol Psychother. 2011;18(1): 75-79.

2. Son KL, Choi JS, Lee J, et al. Neurophysiological features of Internet gaming disorder and alcohol use disorder: a resting-state EEG study. Transl Psychiatry. 2015;5(9): e628.

3. Kuitunen-Paul S, Roerecke M. Alcohol Use Disorders Identification Test (AUDIT) and mortality risk: a systematic review and meta-analysis. J Epidemiol Community Health. 2018;72(9): 856-863.

1. Delorme A, Makeig S. EEGLAB: an open source toolbox for analysis of single-trial EEG dynamics including independent component analysis. J Neurosci Methods. 2004;134(1): 9-21.
2. Wang J, Xu J, Liu S, et al. Electroencephalographic Activity and Cognitive Function in Middle-Aged Patients with Obstructive Sleep Apnea Before and After Continuous Positive Airway Pressure Treatment. Nat Sci Sleep. 2021;13: 1495-1506.

6. Korzeczek A, Neef NE, Steinmann I, et al. Stuttering severity relates to frontotemporal low-beta synchronization during pre-speech preparation. Clin Neurophysiol. 2022;138: 84-96.

7. Wait E, Winter M, Bjornsson C, et al. Visualization and correction of automated segmentation, tracking and lineaging from 5-D stem cell image sequences. BMC Bioinformatics. 2014;15(1): 328.

8. Feldmann LK, Lofredi R, Neumann WJ, et al. Toward therapeutic electrophysiology: beta-band suppression as a biomarker in chronic local field potential recordings. NPJ Parkinsons Dis. 2022;8(1): 44.

9. Požar R, Giordani B, Kavcic V. Effective differentiation of mild cognitive impairment by functional brain graph analysis and computerized testing. PLoS One. 2020;15(3): e0230099.

10. Liao W, Wu GR, Xu Q, et al. DynamicBC: a MATLAB toolbox for dynamic brain connectome analysis. Brain Connect. 2014;4(10): 780-790.
